# Case Report: Isolated surgical decompression for compressive internal jugular vein stenosis: case series and literature review

**DOI:** 10.3389/fsurg.2025.1639108

**Published:** 2025-09-16

**Authors:** Haiyang Ma, Rui Zhao, Shuaibin Lu, Weicheng Peng, Xupeng Peng, Sheng Xu, Beibei Mao, Guangtong Zhu, Zhiqiang Hu

**Affiliations:** ^1^ Department of Neurosurgery, Beijing Shijitan Hospital, Capital Medical University, Beijing, China; ^2^ Department of Neurosurgery, Peking University Ninth School of Clinical Medicine, Beijing, China

**Keywords:** internal jugular vein stenosis, surgical decompression, transverse process of the atlas, venous outflow obstruction, three-dimensional CT venography

## Abstract

**Background:**

Internal jugular vein stenosis (IJVS) is an underrecognized cause of cerebral venous outflow obstruction, commonly presenting with nonspecific symptoms such as head-noise, tinnitus, dizziness, headaches, and visual or auditory disturbances. Extrinsic compression by bony structures, particularly transverse process of the atlas (C1), has emerged as a significant but frequently ignored etiology. This study presents the first case series demonstrating that isolated resection of the C1 transverse process can restore venous outflow and provide durable relief of head-noise–dominant symptoms.

**Objective:**

This study presents three cases of symptomatic IJVS caused by bony compression, marked by head noise–dominant presentation and failure of conservative or endovascular treatments, emphasizing the diagnostic challenges, individualized surgical strategies, and clinical outcomes, along with a review of current literature.

**Methods:**

Three patients with imaging-confirmed compressive IJVS underwent Doppler ultrasound, 3D computed tomography venography, and magnetic resonance imaging. All patients receivedtargeted surgical decompression via resection of the compressive bony structures, with one patient receiving adjunctive venous stenting due to persistent flow limitation.

**Results:**

All patients achieved significant postoperative improvement, including resolution of head noise and amelioration of associated symptoms. Imaging confirmed improved venous caliber and outflow. Notably, one patient with previous stenting failure benefited from staged decompression and re-intervention, highlighting the value of individualized management.

**Conclusion:**

Extrinsic compression is a treatable cause of IJVS. Isolated surgical decompression offers a viable treatment option, particularly in patients unresponsive to endovascular approaches. These cases support the need for greater awareness of compressive IJVS and further studies to refine treatment indications and evaluate long-term outcomes.

## 1 Introduction

Internal jugular vein stenosis (IJVS), a subtype of cerebral venous return disorders, is characterized by a constellation of symptoms including head noise, tinnitus, dizziness, hearing loss, visual impairment, and sleep disorders (1). The internal jugular vein (IJV) serves as the primary conduit for cerebral venous outflow, with approximately 79% of cerebral blood flow exiting through the IJV after drainage from the cerebral and cerebellar veins, while the remaining 21% bypasses via venous plexuses into the external jugular system (2). Consequently, IJVS may impede cerebral venous return, resulting in intracranial hypertension, disturbed cerebrospinal fluid (CSF) dynamics, and stagnation of metabolic waste products. Etiologically, IJVS result from intraluminal haemodynamic abnormalities, structural venous malformations, or extrinsic compression (3). Among these, stenosis caused by adjacent bony structures, most commonly the transverse process of the atlas (C1), is increasingly recognized yet remains underreported.

Diagnostic evaluation relies on ultrasonography, computed tomography venography (CTV), and digital subtraction angiography (DSA) to determine the underlying mechanism and assess the severity of stenosis. Existing management strategies primarily involve conservative measures, surgical decompression, and endovascular stenting, yet debates persist regarding their efficacy, complication profiles, and long-term outcomes. Conservative measures are often ineffective, while endovascular stenting carries a high risk of restenosis when the extrinsic compressive source remains unaddressed. Surgical decompression, though theoretically promising, is rarely detailed in the literature. To date, no published series has systematically reported outcomes following isolated resection of the C1 transverse process in patients with predominant head-noise symptoms who have failed prior conservative or endovascular therapies. In this article, we present three such cases of compressive IJVS, highlighting their clinical features, diagnostic challenges, and surgical management. By detailing the technical approach and outcomes, we aim to provide practical insights into the treatment of IJVS caused by bony compression.

## 2 Case presentation

### 2.1 Case 1

A 46-year-old female, complaining of head noise for more than 3 months, developed intermittent occipital pain in the month before admission. She had previously suffered from a malignant tumour of the left breast, which did not recur on regular review after surgery. Neurological physical examination showed no obvious abnormality.

**Figure 1:**
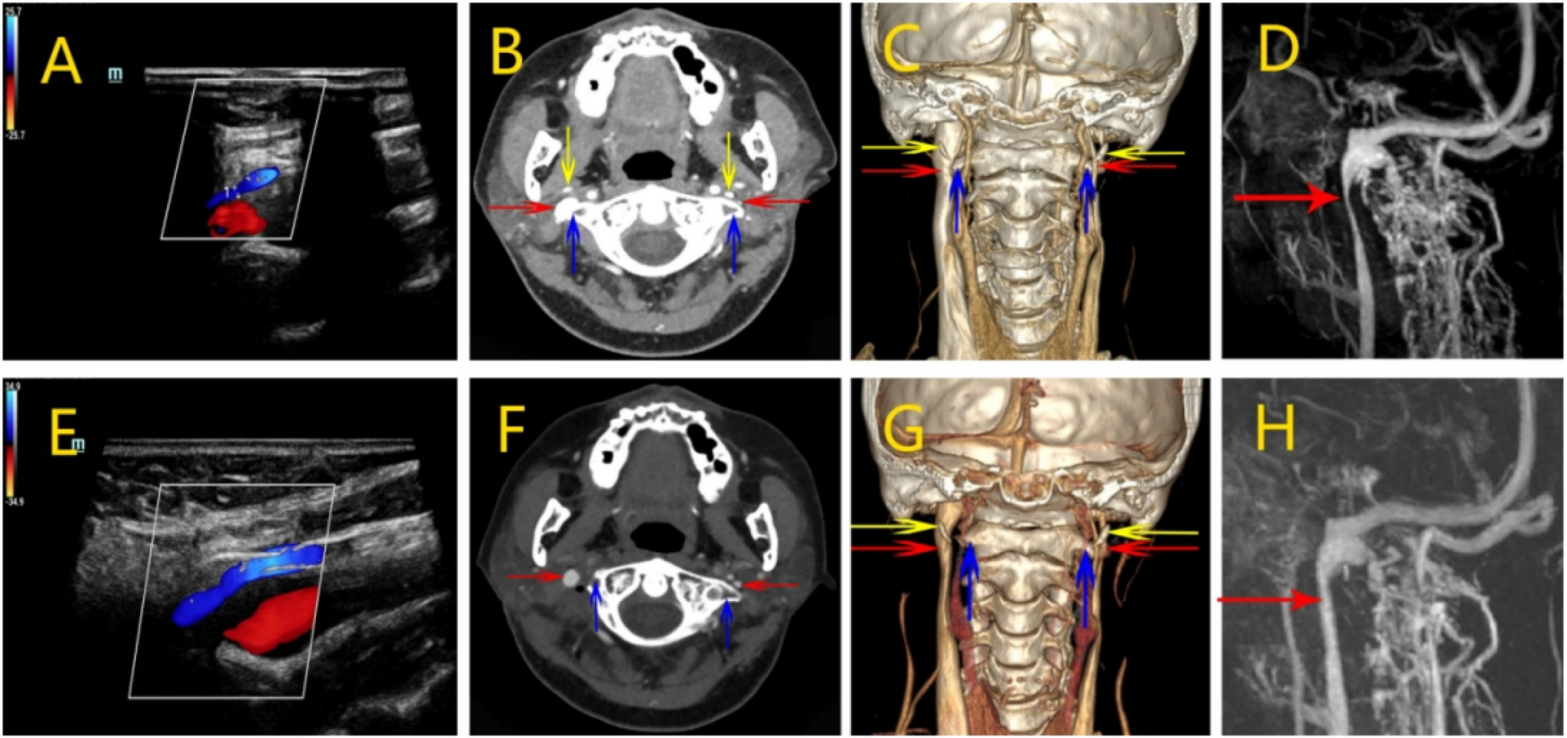
Perioperative imaging analysis of right internal jugular vein decompression in Case 1. **(A)** Preoperative neck ultrasound showed decreased blood flow in the right internal jugular vein. **(B)** Preoperative CTV showed localized compression and stenosis of the J3 segment of the right internal jugular vein. **(C)** Preoperative 3D-CTV showed that the J3 segment of the right internal jugular vein was visibly compressed, despite being the dominant side. **(D)** Preoperative MRV showed a significant narrowing of the right internal jugular vein superiorly located between the right atlantoaxial vertebrae and the right styloid process. **(E)** Postoperative ultrasound showed a slight increase in IJV blood flow. **(F)** Postoperative CTV showed restoration of the right internal jugular vein caliber at the J3 segment with no residual focal stenosis. **(G)** Postoperative 3D-CTV showed the right internal jugular vein is slightly congested compared to the preoperative period. **(H)** Postoperative MR showed relief of right internal jugular vein stenosis. (Red arrow: internal jugular vein, blue arrow: atlas transverse process, yellow arrow:styloid process).

Cranial MR imaging and DWI showed cavernous cerebral infarction next to the posterior horn of the left lateral ventricle. Non-enhanced cranial magnetic resonance venography (MRV) showed localised luminal fibrillation of the left transverse and sigmoid sinuses, which was considered to be a congenital variant. Preoperative vascular ultrasound of the neck showed that the internal diameter of the J1 segment of the left internal jugular vein was 7.5 mm, with a blood flow of 41 ml/min; the internal diameter of the J2 segment was 2.7 mm, with a blood flow of 10.1 ml/min; and the internal diameter of the J3 segment was 1.3 mm, with an undetectable blood flow. The J1 segment of the right internal jugular vein had an internal diameter of 10.3 mm, with a blood flow of about 431 ml/min; the J2 segment had an internal diameter of 4.3 mm, with a blood flow of about 167 ml/min; and the J3 segment had an internal diameter of 4.3 mm, with a blood flow of about 643 ml/min (Figure 1A). Axial computed tomography (CTV) images showed localized compression and narrowing of the J3 segment of the right internal jugular vein (Figure 1B). Three-dimensional CT venography(3D-CTV) further confirmed visible compression of the J3 segment of the right IJV despite it being the dominant drainage pathway (Figure 1C). MRV demonstrated significant narrowing of the J3 segment of the right IJV superiorly located between the right atlantoaxial vertebrae and the styloid process (Figure 1D).

**Figure 2:**
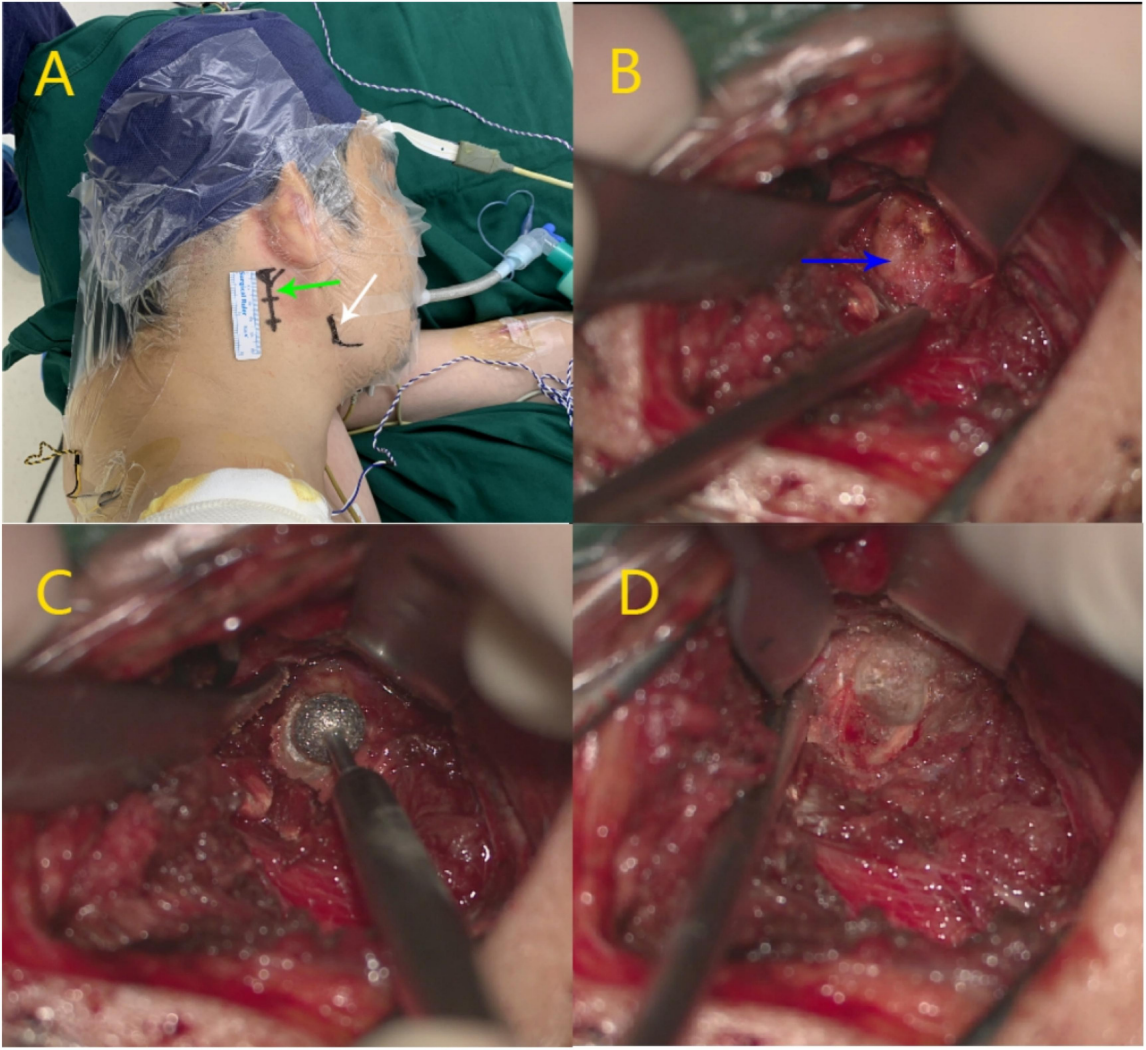
Surgical steps of atlas transverse process resection. Surgical incision of atlas transverse process resection. **(A)** 3–4 cm vertical incision was made along the longitudinal axis, centered just below the mastoid tip. **(B)** Expose the transverse process of the atlas. **(C)** Grind away the transverse process of the atlas. **(D)** Completely grind away the transverse process of the atlas. (Green arrow: the transverse process of the atlas, white arrow: the mandibular angle).

After obtaining informed consent, surgical decompression of the right internal jugular vein was performed via partial resection of the right atlas transverse process. The surgical procedure was as follows: a 3–4 cm vertical skin incision was made along the longitudinal axis, centered just below the mastoid tip (Figure 2A). After careful dissection through the subcutaneous tissue and muscle layers, the transverse process of the atlas was identified and exposed (Figure 2B). High-speed drilling was then applied to gradually grind away the transverse process (Figure 2C), and approximately 7 mm of bone was resected to relieve the compression on the internal jugular vein (Figure 2D). Intraoperative hemostasis was achieved and no neurovascular complications were noted. Postoperatively, the patient did not experience shoulder dysfunction or other motor deficits. However, the subjective symptoms of head noise were not significantly alleviated. For consistency, the surgical procedures of the subsequent two cases were identical and will not be repeated in detail.

On the 5th postoperative day, Neck vascular ultrasound showed that the left internal jugular vein J1 segment internal diameter was 6.6 mm, with a blood flow was about 51 ml/min; J2 segment internal diameter was 2.7 mm, with a blood flow was about 14 ml/min; and J3 segment internal diameter was 1.8 mm, with a blood flow was about 29 ml/min. The right internal jugular vein J1 segment internal diameter 9.7 mm, blood flow about 141 ml/min; J2 segment internal diameter 5.2 mm, blood flow about 108 ml/min; J3 segment internal diameter 3.1 mm, blood flow about 133 ml/min. Blood flow in the right internal jugular vein was slightly increased from the preoperative period (Figure 1E). CTV and 3D-CTV that the caliber of the right IJV at the J3 segment had been restored with no residual focal stenosis (Figures 1F,G). Postoperative MRV demonstrated a substantial improvement in the morphology of the right internal jugular vein, particularly at the J3 segment where prior compression was noted. The vessel lumen appeared smooth and continuous without residual narrowing, suggesting effective decompression following partial resection of the right atlas transverse process (Figure 1H). On follow-up 12 months after the operation, the patient complained of significant relief of head noise symptoms.

### 2.2 Case 2

A 61-year-old female, complained of head noise in the right ear for more than 15 years, accompanied by head noise, dizziness and headache for 2 years. She was referred to the Otolaryngology Department of an outside hospital for hearing loss on the left side, and was treated symptomatically with medication. Comorbidities included anxiety disorder. There were no positive signs on neurological physical examination.

**Figure 3:**
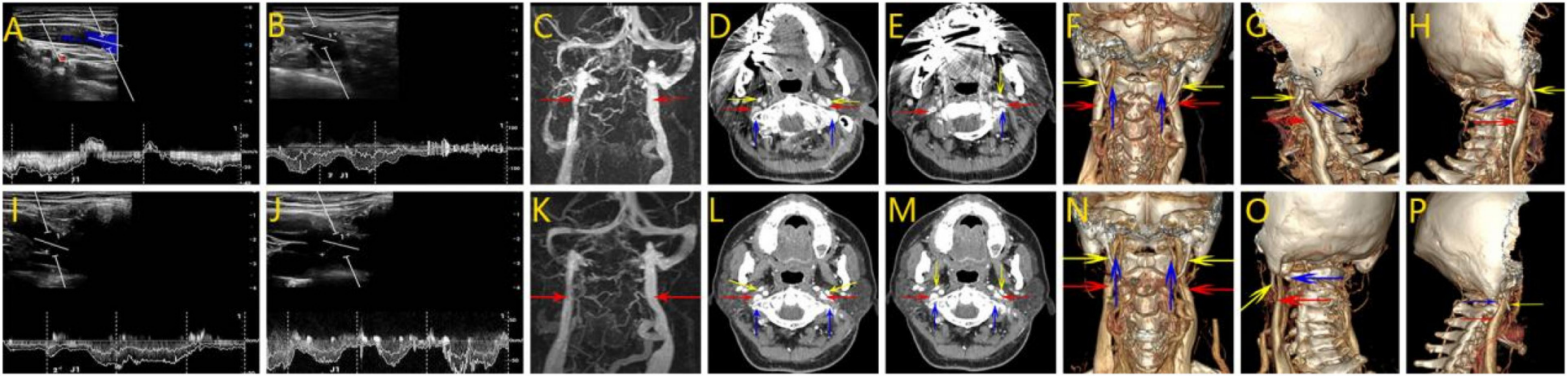
Perioperative imaging analysis of left internal jugular vein decompression in Case 2. **(A,B)** Preoperative neck ultrasound of the right and left internal jugular veins, respectively. The contrast shows a narrower J1 segment of the left internal jugular vein with reduced blood flow. **(C)** MR of the neck showed a significant narrowing of the left internal jugular vein superiorly located between the left atlantoaxial vertebrae and the left styloid process. **(D,E)** The CTV image shows localized compression and stenosis of the J3 segment of the internal jugular veins bilaterally, with balanced transverse and sigmoid sinuses. **(F–H)** 3D-CTV images of the frontal, left and right positions, respectively. There is slight compression and narrowing of the J3 segment of the internal jugular vein bilaterally. **(I,J)** Postoperative ultrasonographic findings of the J1 segment on both sides, respectively. The diameter of the J1 segment of the left internal jugular vein was slightly increased. **(K)** Postoperative MR. Relief of left internal jugular vein stenosis. **(L,M)** Postoperative CTV showed improved stenosis. **(N–P)** 3D-CTV showed that the left internal jugular vein was slightly less compressed after the left operation than before. (Red arrow: internal jugular vein, blue arrow: atlas transverse process, yellow arrow:styloid process).

After admission, otolaryngology and psychiatry were asked to assist in the diagnosis and to rule out related diseases causing head noise. Stereoscopic blood flow examination of the jugular vein showed that the left internal jugular vein J1 segment had an inner diameter of 8.7 mm, with a blood flow of about 244 ml/min (Figure 3A); the J2 segment had an inner diameter of 6.6 mm, with a blood flow of about 153 ml/min; the J3 segment had an inner diameter of 3.9 mm, with a blood flow of about 116 ml/min. The right internal jugular vein J1 segment had an inner diameter of 9.5 mm, with a blood flow of about 898 ml/min (Figure 3B); the J2 segment had an inner diameter of 7.8 mm, with a blood flow of about 7.8 mm, and the J2 segment had an inner diameter of about 898 ml/min. 7.8 mm, blood flow about 400 ml/min; J3 segment inner diameter 5.4 mm, blood flow about 288 ml/min. Left internal jugular vein blood flow is reduced. MR enhancement imaging of the neck showed that the upper end of the left internal jugular vein travelled between the left atlantoaxial vertebra and the left styloid process, and the outer and posterior edges of the vein were compressed in the shape of a sharp angle (Figure 3C). Admission axial computed tomography angiography (CTV) images showed localised compression and narrowing of the J3 segment of the internal jugular vein bilaterally, with balanced transverse and sigmoid sinuses (Figures 3D,E). The 3D-CTV image of bone remodelling showed that the J3 segment of the internal jugular vein bilaterally travelled locally between the transverse process of the atlantoaxial vertebrae and the styloid process and was seen to be slightly compressed and narrowed. The left internal jugular vein was compressed and fibrous at the level of the thoracic inlet (Figures 3F–H).

After admission, the patient's bilateral transverse sinus and sigmoid sinus were balanced, and the diagnosis of bilateral internal jugular vein stenosis was clear, with the left side being heavier. After evaluating the patient's examination results, there were no contraindications to surgery, and after obtaining the consent of the patient and his family, the left atlantoaxial transverse process was partially abraded under general anaesthesia to decompress the internal jugular vein. The left atlantoaxial transverse process was removed under microscope, especially the bone crest protruding anteriorly and inferiorly from the transverse process, and the compressed internal jugular vein was sufficiently decompressed. The extent of abrasion was about 1.8 cm, and the decompression effect was satisfactory. The patient complained of numbness of the left upper limb after the operation, and the cause of the surgical pull was considered. Symptoms such as head noise were seen to be significantly relieved.

Postoperative stereoscopic blood flow examination of the jugular vein showed that the internal diameter of the left internal jugular vein was 9.1 mm in J1 segment, 7.5 mm in J2 segment, and 4.9 mm in J3 segment (Figure 3I); the internal diameter of the right internal jugular vein was 11.6 mm in J1 segment, 7.8 mm in J2 segment, and 5.9 mm in J3 segment (Figure 3J). The blood flow in the left J1 segment was about 316 ml|min, J2 segment was about 73 ml|min, and J3 segment was about 132 ml|min; the blood flow in the right J1 segment was about 1,283 ml|min, J2 segment was about 316 ml|min, and J3 segment was about 343 ml|min.MR showed that the left internal jugular vein stenosis was relieved compared with the preoperative period (Figure 3K). Postoperative CTV showed stenosis was better than before (Figures 3L,M). CT 3D imaging of the jugular vein was repeated on postoperative day 7 and showed a localized bony defect in the left transverse process with slight swelling of the surrounding soft tissues. The left internal jugular vein at the thoracic inlet was compressed and slightly more elongated than before (Figures 3N–P). By postoperative day 9, the patient reported marked improvement in head noise. At the 4-month follow-up, the symptom had further subsided and was nearly resolved.

### 2.3 Case 3

A 52-year-old male complained of paroxysmal headaches for 6 years and was treated symptomatically with medications in an outside hospital. Comorbidities included hypertension and a history of cardiac pacemaker implantation, which precluded MRV. Neurologic examination showed no significant positive signs.

**Figure 4:**
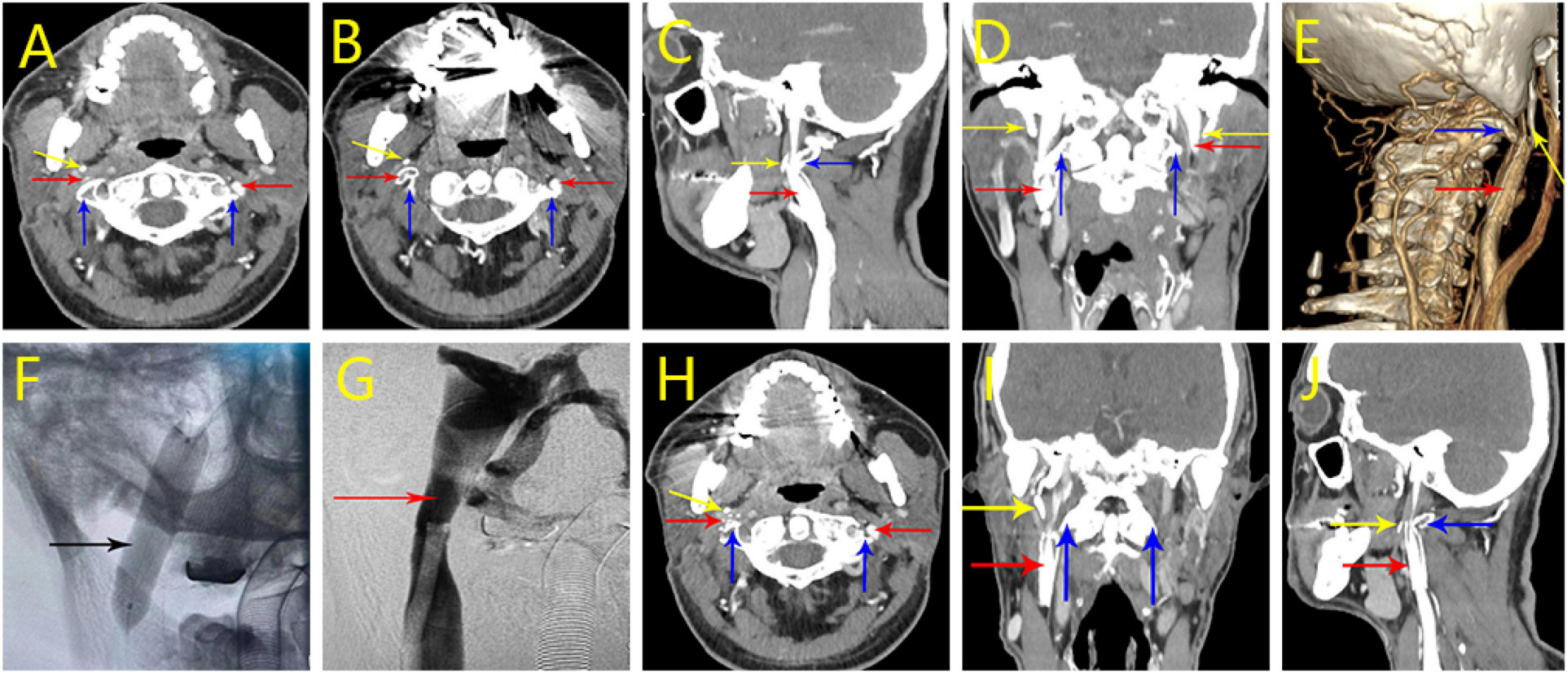
Perioperative imaging analysis of hybrid surgical-interventional approach in Case 3. **(A–D)** CTV on initial admission. stenting of the right jugular vein with stent lumen narrowing and stenosis of the right jugular vein at the C1 level. **(E)** 3D-CTV on new admission. Right side stenosis is due to bone compression. **(F)** Postoperative stent expansion was favorable. **(G)** Postoperative angiography showed improved luminal stenosis and vessel patency. **(H–J)** Postoperative CTV results. It shows that after the right operation, the right internal jugular vein lumen stenosis has improved compared to the preoperative period. (Red arrow: internal jugular vein, blue arrow: atlas transverse process, yellow arrow: styloid process, black arrow: internal jugular vein stent).

The patient was diagnosed with bilateral jugular vein stenosis 3 years ago at an outside hospital and underwent stenting of the right internal jugular vein, which provided a slight relief of symptoms after the procedure but recurred several months later. Axial computed tomography angiography (CTV) images (Figures 4A–D) performed after the patient was admitted to the hospital showed in-stent thrombosis in the stent, restenosis of the stent lumen, and stenosis of the C1 transverse segment of the right jugular vein after stenting of the right jugular vein. The 3D-CTV image of bone remodeling (Figure 4E) showed the right stenosis as bony compression.

Upon admission and completion of the examination, the patient was diagnosed with bilateral internal jugular vein stenosis. No contraindications to surgery were seen after evaluating the patient's examination results, and after obtaining the consent of the patient and his family, a complex surgery was performed under general anesthesia. First, the right atlantoaxial transverse process was partially abraded to decompress the internal jugular vein, and the abraded transverse process was about 5 mm, and the decompression effect was satisfactory. Subsequently, total cerebral angiography and balloon dilatation of the right internal jugular vein stent were performed, and postoperative angiography showed improvement of luminal stenosis and patency of the vessel (Figures 4F,G).

On the 4th day after the operation, the jugular vein CT three-dimensional imaging was repeated, and the examination results showed partial bony defects in the localized right transverse process, and the surrounding soft tissues were slightly swollen. The stenosis of the right internal jugular vein lumen improved compared with the preoperative period (Figures 4H–J). At the time of discharge, the patient complained of slight relief of headache symptoms. On return visit 1 year postoperatively, the patient's headache symptoms had improved from before.

## 3 Literature review

To assess the current evidence, we conducted a PubMedMEDLINE literature review using the search terms: [(compressive internal jugular vein stenosis) OR (IJVS)] AND [(treatment)OR (Surgical treatment)] on June 30th, 2025. Inclusion criteria were: (a) human studies, (b) reporting compressive internal jugular vein stenosis treatment, (c) invasive treatment. 10 of the 121 records met these criteria, and the data are summarized in Tables 1, 2 (4–13). The studies included ranged from case reports to large single-center cohort studies. In all the above studies and cases, a total of 248 patients underwent invasive treatment, most of whom underwent stenting. A subset of patients underwent decompression surgery (styloidectomy or C1 resection), but these patients largely underwent stenting at the same time. In the majority of reported cases, patients who underwent invasive treatment experienced improvement in their symptoms. Some post-interventional patients experienced symptomatic recurrence and underwent decompression surgery, and the efficacy of the decompression surgery was stable. Only a few of the 248 patients underwent decompression surgery alone, which was mainly presented as case reports, and most of the surgical procedures involved were performed with styloidectomy. C1 resection was also used in clinical practice, but basically in combination with stent implantation or styloidectomy, and C1 resection alone was not widely used in the previously reported case surgery.

**Table 1:** Surgical treatment of internal jugular vein stenosis reported in case reports and case series.

Author	Total patients	Surgical patients	Surgical indication	Invasive treatment	Outcomes	Study type
Ahsan et al. (4)	1	1	Thoracic inlet syndrome.	Enlargement of manubrium and sternum.	Relief of symptoms.	Case report
Dashti et al. (5)	2	2	Compression by styloid process.	One patient underwent styloidectomy,1 patient underwent styloidectomy, C1 resection and stenting.	Relief of symptoms, IJV pressure gradient drop.	Case series
Li et al. (6)	1	1	Compression by C1 lateral mass and styloid process.	Left underwent styloidectomy, C1 resection and stenting,right underwent stenting.	Poor results with unilateral surgery, symptomatic relief with bilateral surgery.	Case report
Fritch et al. (7)	1	1	Compression by C1 lateral Mass.	C1 resection and stenting.	Relief of symptoms.	Case report
Yang et al. (8)	14	14	Compression by C1 lateral mass and styloid process.	14 underwent styloidectomy and C1 resection,2 patients underwent stenting.	Significant improvement of IJV stenosis in eleven patients and mild improvement in three.	Case series

19 surgically managed patients were reported in case reports or small series. Main indications were thoracic inlet syndrome, styloid process compression, and C1 lateral-mass compression; interventions included styloidectomy, C1 resection, and jugular venous stenting, alone or combined, with symptomatic improvement reported in most cases.

**Table 2:** Surgical treatment of internal jugular vein stenosis reported in cohort studies.

Author	Total patients	Surgical patients	Surgical indication	Invasive treatment	Outcomes	Study type
Zhou et al. (9)	15	15	Isolated IJVS	Stenting	Relief of symptoms,IJV pressure gradient drop.	Retrospective study
Bai et al. (10)	27	1	Compression by styloid process.	One patient underwent styloidectomy, C1 resection and stenting.	Symptom reduction at follow-up.	Prospective study
Ding et al. (11)	46	1	Compression by C1 lateral mass and styloid process, Patient self-request.	One patient underwent styloidectomy, C1 resection and stenting.	Symptom reduction at follow-up.	Prospective study
Bai et al. (12)	183	183	Compression or unknown cause.	Stenting	Improvement of symptoms, especially headaches.	Retrospective study
Fargen et al. (13)	29	29	Compression	Some patients underwent stenting after styloidectomy and C1 resection	Partial symptomatic improvement with complications	Retrospective study

229 surgically managed patients were reported from cohort studies. Indications were styloid or C1 lateral-mass compression. Stenting was the primary treatment, with selective decompression by styloidectomy and/or C1 resection. Follow-up commonly showed symptom relief and lower jugular venous pressure gradients. Complications were uncommon.

## 4 Discussion

Internal jugular vein stenosis (IJVS) can be broadly categorized into compressive and non-compressive types based on its etiology. Compressive IJVS arises due to external anatomical structures exerting pressure on the internal jugular vein, frequently identified as the primary cause in clinical imaging studies (3). Common anatomical sources of compression include lymph nodes, the thyroid gland, the carotid artery, fascial bands, stenotic jugular foramina, and notably, the atlantoaxial transverse process (6, 14). In contrast, non-compressive IJVS typically results from intrinsic structural abnormalities of the vein itself, such as fused, ectopic, or inverted venous valves, which directly impede venous flow (15).

Regardless of etiology, IJVS results in obstruction of cerebral venous return, initiating a cascade of pathophysiological alterations. These changes include abnormalities in cerebral hemodynamics (16), compromised integrity of the blood-brain barrier (17), disturbances in cerebrospinal fluid (CSF) dynamics (18), cerebral white matter damage, and elevated intracranial pressure (9). Clinically, head noise (unilateral or bilateral) is a common and characteristic presentation of IJVS (19). We currently hypothesize that head noise may result from alterations in cerebral hemodynamics. Patients presenting with atypical symptoms—such as dizziness, headaches, tinnitus, sleep disturbances, hearing loss, anxiety, and depression—often seek consultation across various specialties, including otolaryngology, ophthalmology, and psychiatry, without achieving substantial relief.

Recently, the incidence of compressive internal jugular vein stenosis (IJVS) diagnoses has risen significantly; however, standardized guidelines for diagnosis and evaluation are still lacking. Patients are typically diagnosed with IJVS after excluding arterial system diseases and other underlying causes. Currently, diagnostic assessment of IJVS primarily relies on vascular ultrasonography, CT venography (CTV), and angiography (20–22). Ultrasonography accurately measures vein diameters and evaluates blood flow dynamics, providing essential hemodynamic insights. CTV clearly demonstrates anatomical relationships between the internal jugular vein and surrounding structures, particularly bony compressions. Angiography remains the gold standard, precisely identifying stenosis location, severity, and associated pressure gradients, thus guiding surgical or interventional decision-making (23, 24).

Current treatment strategies for IJVS vary depending on etiology and compressive structures. Among the causes of internal jugular vein stenosis (IJVS), common bony structures include the atlantoaxial transverse process, the pedicle, and narrowed jugular foramina. For internal jugular vein stenosis caused by compression from bony structures, the compression can be relieved by grinding away the compressed bone tissue. After grinding the bone, adequate release of the surrounding soft tissues can further relieve the patient's symptoms. Intraoperatively, the internal jugular vein diameter was seen to be significantly inflated, and postoperative follow-up showed that the patients' symptoms were significantly improved. For internal jugular vein stenosis caused by the compression of soft tissue structures such as the diastasis, sternocleidomastoid muscle, and carotid artery, it is usually preferred to decompress the surrounding tissue on the dominant side of the vein.

The three cases discussed demonstrate different clinical scenarios and treatment choices. In Case 1, the patient had left-sided internal jugular vein stenosis caused by developmental anomalies, and the dominant side of venous return to the head and neck was on the right side. Abrasion of the left atlas alone could not improve the venous return on the left side, so after a comprehensive evaluation of the patient's condition, release surgery on the right side was chosen to increase the blood flow in the veins of the head and neck. Case 2 patient was definitively diagnosed with bilateral internal jugular vein stenosis on preoperative examination, and the transverse sinus and sigmoid sinus were in equilibrium bilaterally. According to the internal jugular vein Doppler ultrasound findings, the blood flow in the left side of the vessel was significantly reduced, and the degree of stenosis was severe. Therefore, atlantoaxial partial abrasion decompression surgery was selected for the left side, and the patient's symptoms were significantly relieved after the surgery. In a more complex scenario, Case 3 presented prior unsuccessful stenting due to persistent bone compression. A hybrid approach—combining surgical resection of the right atlantoaxial transverse process with endovascular stent revision—achieved durable luminal expansion and symptom resolution, underscoring the value of surgical decompression and interventional techniques for complex compressive IJVS.

Reviewing the literature, surgical decompression for IJVS is often reported in combination with stenting, with fewer cases detailing isolated surgical interventions. Our case series demonstrates that isolated surgical decompression can achieve significant symptomatic relief and improved hemodynamic parameters, offering an essential alternative to patients not suited for or failing stenting procedures. Furthermore, the selective removal of the transverse process rather than the more commonly reported styloidectomy represents a noteworthy variation in surgical strategy, broadening the surgical repertoire for compressive IJVS.

## 5 Conclusion

IJVS can cause a disabling constellation of symptoms, such as intrusive head noise/tinnitus, positional headache, cognitive fatigue, and sleep disturbance, which substantially reduce quality of life and require timely recognition to avoid misdiagnosis and ensure effective treatment. In this case series of three patients, isolated resection of the C1 transverse process enhanced venous caliber and outflow on imaging, with clinical outcomes ranging from complete symptom resolution to partial improvement. This strategy also served as a salvage option when stenting alone failed because of persistent bony conflict. In the absence of standardized diagnostic pathways, larger prospective studies are warranted to refine indications, evaluate long-term durability, and optimize patient selection for isolated decompression vs. hybrid approaches.

## Data Availability

The original contributions presented in the study are included in the article/Supplementary Material, further inquiries can be directed to the corresponding authors.
